# Supplementary figures and images for: Emergence of visible light optical properties of L-phenylalanine aggregates
Source: PeerJ. 2019 Feb 25;7:e6518. doi: 10.7717/peerj.6518 (PMC6394350; doi:10.7717/peerj.6518)

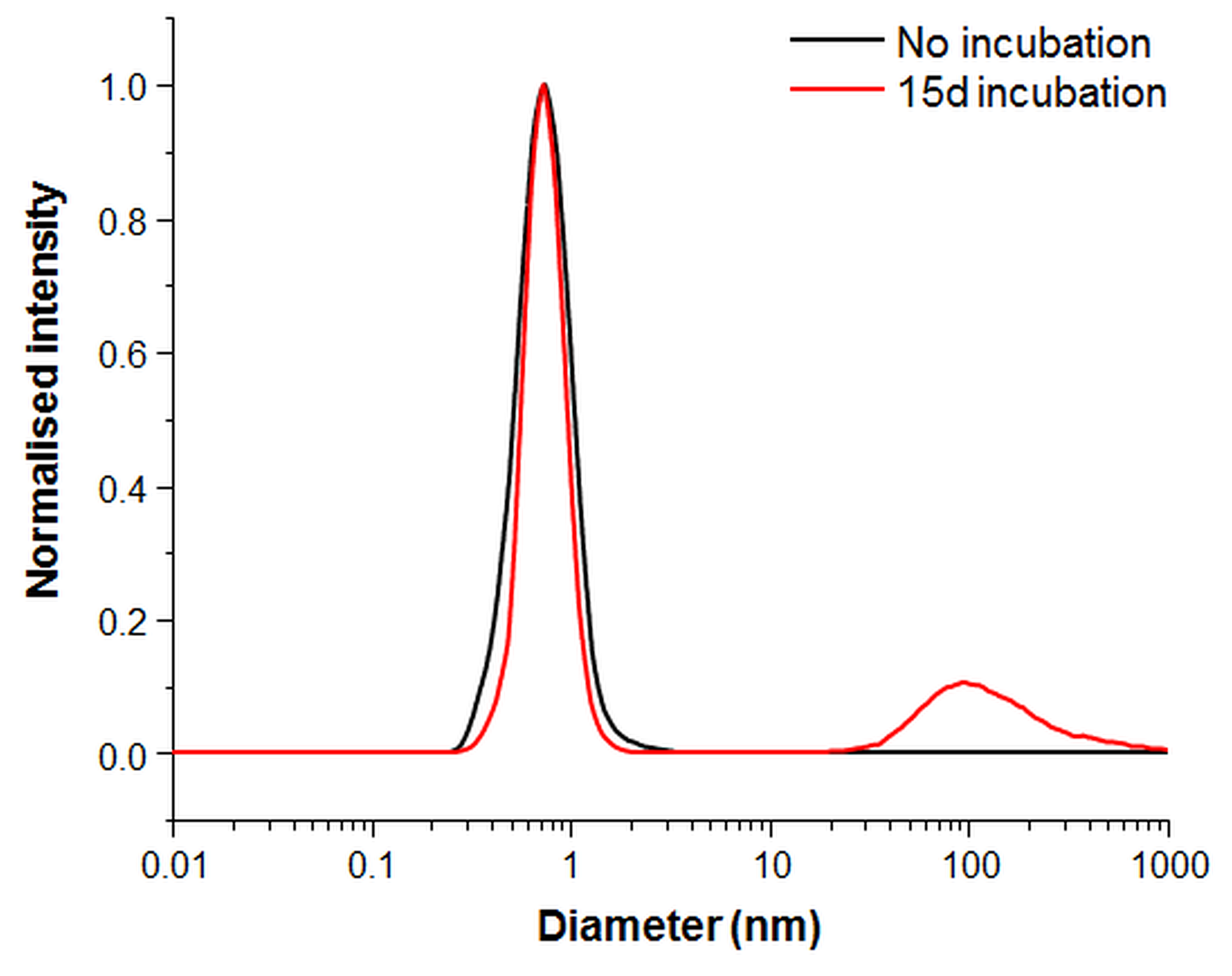

Supplement: Supplemental Information 2 — The size measurements were performed at 25 °C using Malvern Zetasizer μV. For each sample 10 repeats of 10 scans were recorded. [file peerj-07-6518-s002.png]

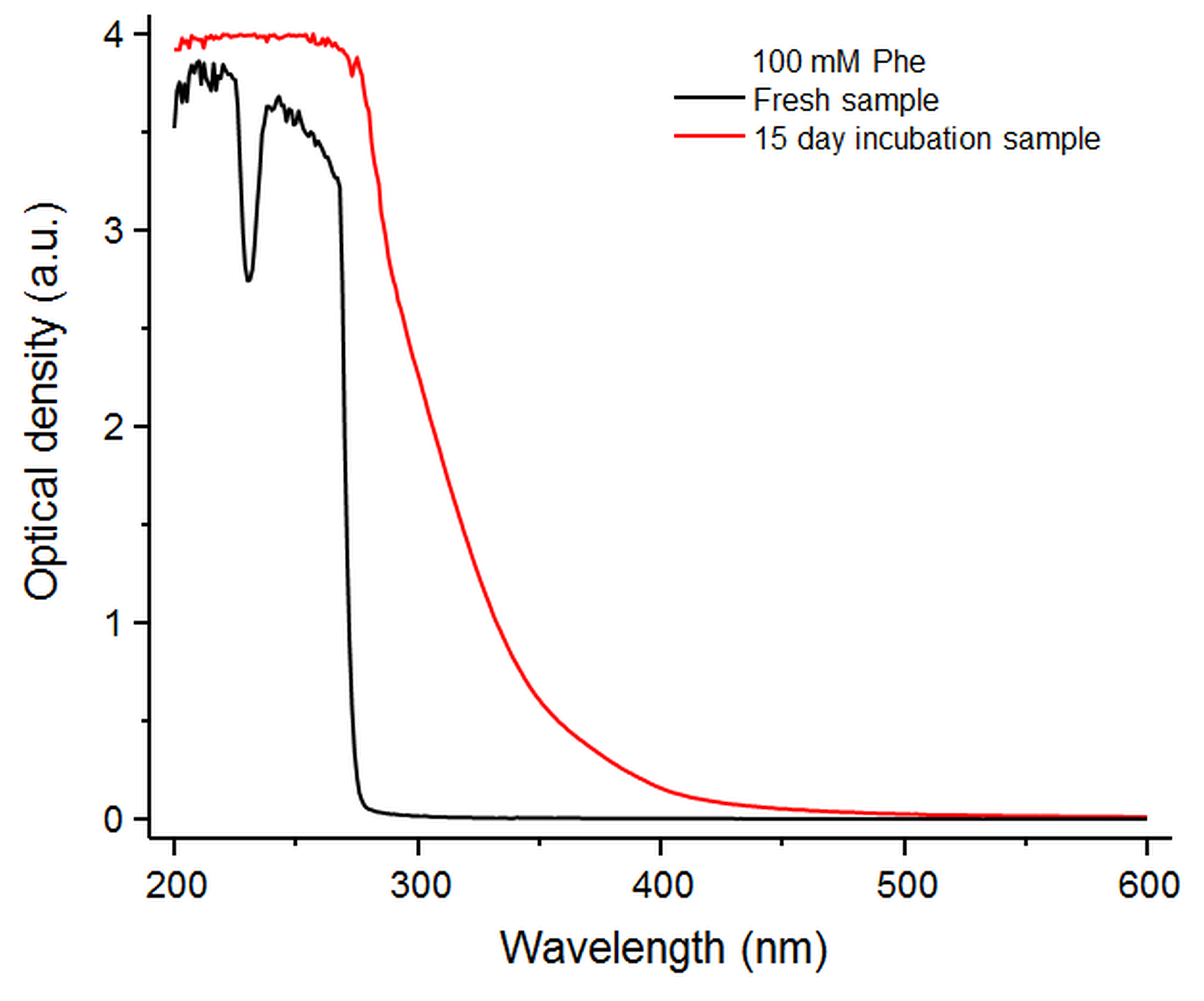

Supplement: Supplemental Information 3 [file peerj-07-6518-s003.png]
